# Benefits of Dietary Supplementation with Specific Silicon-Enriched Spirulina on Arterial Function in Healthy Elderly Individuals: A Randomized, Placebo-Controlled Trial

**DOI:** 10.3390/nu17050864

**Published:** 2025-02-28

**Authors:** Anne Virsolvy, Amir Mokhfi Benmira, Salim Allal, Christophe Demattei, Thibault Sutra, Jean-Paul Cristol, Nicolas Jouy, Sylvain Richard, Antonia Perez-Martin

**Affiliations:** 1 PhyMedExp, Université de Montpellier, INSERM U1046, CNRS UMR 9214, 34295 Montpellier, France; jp-cristol@chu-montpellier.fr (J.-P.C.); sylvain.richard@inserm.fr (S.R.); 2 Department of Vascular Medicine, Nîmes University Hospital, Université de Montpellier, 30900 Nîmes, France; benamira.amir@gmail.com (A.M.B.); salim.allal@chu-nimes.fr (S.A.); antonia.perez.martin@chu-nimes.fr (A.P.-M.); 3 BESPIM–Laboratoire de Biostatistique, Epidémiologie Clinique, Santé Publique Innovation et Méthodologie, Nïmes University Hospital, Université de Montpellier, 30900 Nîmes, France; christophe.demattei@chu-nimes.fr; 4 Department of Biochemistry, Montpellier University Hospital, 34295 Montpellier, France; t-sutra@chu-montpellier.fr; 5 Phyco-Biotech, 34000 Montpellier, France; n.jouy@phyco-biotech.com; 6 IDESP-Institut Desbrest D’Epidémiologie et de Santé Publique, Université de Montpellier, INSERM U1318, 34090 Montpellier, France

**Keywords:** spirulina, silicon, aging, arterial function, blood pressure, pulse wave velocity

## Abstract

*Background/Objectives*: Vascular aging is associated with increased arterial stiffness and changes in the wall structure, leading to a loss of elasticity. Silicon is abundant in arteries and plays a key role in the synthesis and stabilization of elastin fibers. In animal models of accelerated cardiovascular aging, a specific nutritional supplement based on silicon-enriched spirulina (SpSi) has been shown to have beneficial effects on vascular function. The present study, designed as a randomized, double-blind, placebo-controlled trial, aimed to evaluate the effectiveness of this SpSi supplement on aging-related changes in vascular function among healthy older adults. *Methods*: Here, 120 healthy volunteers aged 60–75 years were enrolled and randomly assigned to either the SpSi group (*n* = 60) or placebo group (*n* = 60). Over 6 months, the participants received either 3.5 g of specific 1% silicon-enriched spirulina (SpSi group) or placebo tablets daily. The primary outcome was the assessment of arterial wall pressure waveforms, which included blood pressure (BP) readings and the determination of the aortic pulse wave velocity (aPWV). Secondary outcomes included the vasomotor endothelial function through post-ischemic vasorelaxation, measured using the reactive hyperemia index (RHI), and carotid intima–media thickness. *Results*: When considering the entire sample, none of the studied parameters differed between the placebo and SpSi groups. However, when focusing on individuals with high–normal blood pressure (i.e., systolic BP between 130 and 150 mmHg) and aPWV levels above cutoff values (>10 m/s), the BP decreased by 8% (*p* < 0.001) and aPWV decreased by 13.5% (*p* < 0.0001) in subjects receiving SpSi. In individuals with BP and aPWV levels below the cutoff values, no effect was observed. *Conclusions*: In healthy elderly individuals, SpSi supplementation improved high–normal blood pressure and aortic pulse wave velocity, suggesting an enhanced vascular function.

## 1. Introduction

Promoting healthy aging represents a significant socio-economic challenge, primarily focused on prevention. Biological aging involves a gradual decline in the structure and function of organs or tissues, leading to various chronic diseases. Notably, aging is an important and independent risk factor for cardiovascular issues. As a result, cardiovascular diseases are among the leading causes of morbidity and mortality in the older population [1]. Cardiovascular aging, involving the deterioration of the arterial structure and function over time [2], is characterized by changes in the elasticity of arterial walls and heart muscle, which includes increased degradation of elastin and the accumulation of abnormal collagen [3]. Macromolecular structures are susceptible to changes that also contribute to various signs of aging, such as wrinkles, sagging tissue, and reduced joint flexibility in addition to the increased risk of cardiovascular disease. The disorganization and progressive degradation of elastic fibers may lead to age-related remodeling of the arteries, particularly affecting the aorta [4], which contains 30 to 40% elastin in its wall [3]. This physiological remodeling, known as arteriosclerosis, is characterized by the thickening of the arterial wall and increased arterial stiffness [5]. As a result, cardiovascular hemodynamics are altered, leading to an increase in central blood pressure. This remodeling may also affect the resistance of peripheral arteries, contributing to further increases in central vascular stiffness and blood pressure (BP) [6,7]. Increased stiffness of the aortic arterial wall is indicated by a rise in the aortic pulse wave propagation velocity, also known as pulse wave velocity (aPWV) [2]. Along with high blood pressure, this increased stiffness is one of the signs of cardiovascular aging. In older adults, these factors are associated with cardiovascular events and a heightened risk of mortality [8,9]. In clinical practice, the measurement of pulse wave velocity (PWV) is generally accepted as the most simple, non-invasive, robust, and reproducible method with which to determine arterial stiffness [10]. It reflects arterial stiffness and increases with the loss of elastic fibers or the rise of collagen fibers. Both age and BP are significant factors influencing aortic pulse wave velocity (aPWV), a marker of arterial stiffness validated as a predictor of cardiovascular events, particularly in seniors [11]. While aging is a natural process, lifestyle factors, such as diet, exercise, and skin care, can affect how quickly and significantly these changes occur. Additionally, certain interventions, like topical treatments, dietary supplements, and medical procedures, may help mitigate some of the effects of aging [12,13,14]. Among them, silicon has attracted particular interest.

Silicon is an essential trace element for human health [15]. It plays a vital role in the formation of bones, connective tissues, and skin [16,17]. Common dietary sources include grains, fruits, and vegetables. However, nutritional intake may become insufficient. The limited bioavailability of silicon can lead to deficiencies, which have been associated with negative health effects [18]. While silicon is not classified as a vital nutrient for humans, it is important for the structure and stability of elastin fibers, especially in large arteries [19]. Additionally, silicon helps maintain tissue elasticity by participating in the organization and synthesis of collagen and elastin fibers. Silicon may play a preventive role in the alterations associated with aging, particularly in relation to cardiovascular health. Its involvement in the synthesis of collagen and elastin could help preserve arterial elasticity and flexibility, thereby reducing the risk of arterial stiffness and the cardiovascular complications that come with it [20]. Various studies conducted on both human and animal models show that dietary silicon enhances the structural integrity of connective tissue and reduces the risk of atherosclerosis [21,22,23,24,25]. However, the bioavailability and absorption of silicon vary significantly based on its chemical form. For instance, dietary supplementation with specific silicon-enriched spirulina has been shown to prevent vascular dysfunction in hamsters fed a high-fat diet [26] and to lower blood pressure in a rat model of hypertension [27]. These positive effects on blood vessels are partially attributed to the silicon metabolized in spirulina but may also involve the beneficial role of spirulina. Spirulina is regarded as a functional food due to its high protein and vitamin content, along with its antioxidant and anti-inflammatory properties [28,29]. Additionally, it has been shown to be effective in promoting vascular function [30,31]. No systemic or liver adverse effects or toxicity were observed in rats, supporting the safety of this dietary supplement [32].

Considering the biological role of silicon (Si) and the beneficial effects of Si-enriched spirulina (SpSi), we assessed the impact of six months of dietary supplementation with SpSi on the morphological, biomechanical, and functional properties of the arterial wall in elderly individuals. The aPWV serves as a marker of arterial stiffness, which tends to increase when BP exceeds normal physiological values and when arterial compliance decreases. Changes in these parameters reflecting vascular function were considered the primary outcome of our study.

## 2. Materials and Methods

### 2.1. Subjects

In this study, 120 healthy volunteers, aged 60–75 y, without any pre-existing chronic illness, treatment, or history of cardiovascular events, were included. The exclusion criteria were as follows: chronic cardiovascular or metabolic disease (including dyslipidemia and hyperglycemia); any chronic pathology requiring medical treatment; active smoking or drug use; arterial stenosis (>50% in diameter); aneurism; arteriopathy of the lower limbs; and hormone replacement therapy in women.

### 2.2. Study Design

This is a longitudinal, interventional, double-blind, randomized, and placebo-controlled study. Two groups were randomly formed of 60 subjects matched by age, sex, and body mass index, equally divided into men and women. Each group was coded and assigned to either placebo or silicon-enriched spirulina (SpSi) for 6 months. The subjects ingested 5 tablets of the placebo or SpSi daily (3.5 g spirulina containing 35 mg of metabolized silicon). Before inclusion, a medical consultation excluded patients with non-inclusion criteria, and a carbohydrate and lipid biological assessment was conducted to detect unknown metabolic diseases. Selected participants were then subjected to four medical examinations and biological analysis: on the day of inclusion (D0) and after 30, 90, and 180 (D180) days. A comparison of the aortic pulse wave velocity (aPWV) between groups was the primary outcome. Secondary outcomes were variations in the blood pressure (BP), carotid intima–media thickness, and endothelial-related post-ischemic vasorelaxation (RHI).

### 2.3. Ethics

The study was registered at ClinicalTrials.gov (NCT03464760) and approved by the Committee for the Protection of Human Subjects, Sud-Est I. All subjects gave written informed consent before participation in the study. All methods and procedures were conducted following the ethical standards of the Declaration of Helsinki and Good Clinical Practice Guidelines.

### 2.4. Dietary Supplement

Specific silicon-enriched spirulina platensis (SpSi) was produced by Phyco-Biotech (Montpellier, France). According to a patented process (EP2015/057596), spirulina was grown in Zarouk’s medium at 22 °C and pH 10.5 and in the presence of 2 g/L silicon for Si enrichment. Spirulina can assimilate and accumulate elements and nutrients; thus, this process produces spirulina biomass containing silicon metabolized inside the spirulina cells. The fresh biomass recovered via filtration was then washed, pressed under vacuum, quickly dried at 42 °C in a vented dryer, cut, and finely ground. With a final Si content of 1%, the powder was then compacted to make 700 mg tablets. The placebo consisted of 700 mg tablets of green-colored microcrystalline cellulose with 1% sodium copper chlorophyllin (*w*/*w*).

### 2.5. Vascular Parameter Assessments

All participants were caffeine abstinent for 6 h prior to the examination. All vascular measurements were performed after at least 15 min of rest in a supine position in a quiet, temperature-controlled (i.e., 24 °C) room. Only two well-trained operators performed all the vascular measurements in the following order: blood pressure, carotid intima–media thickness (IMT), aortic pulse wave velocity, and peripheral endothelial function.

Brachial systolic, diastolic, and mean blood pressures were recorded using an automatic oscillometric sphygmomanometer calibrated within the last year (Dinamap ProCare 300 system (GE Healthcare, Chicago, IL, USA)) by an experienced operator who was present in the room. BP was first measured in both arms to rule out any side differences (an SBP difference ≥ 20 mmHg). Then, it was measured on the dominant arm, twice and separated by at least 3 min. The two values were averaged.

The carotid intima–media thickness (IMT) and carotid diameter measurements on both the left and right common carotid arteries were achieved using high-resolution vascular ultrasonography (Samsung RS85 Prestige ultrasound system, Samsung Medison Co., Ltd., Hongcheon, Republic of Korea), with a 12 MHz multi-frequency linear probe, as previously described and validated. Briefly, the transducer was placed 2–3 cm proximal to the carotid bifurcation. The IMT (mm) was measured automatically by the software in the region of interest drawn by the operator according to the Mannheim consensus [33], and the arterial diameter was measured at the same site.

The aortic pulse wave velocity (aPWV) reflecting aortic stiffness was evaluated through the carotid-to-femoral pulse wave velocity, expressed in meters/sec, between the two sites via the foot-to-foot velocity method using a validated, automatic device (Complior^®^ SP Artech Medical, Pantin, France), as previously described [34,35]. Briefly, aPWV assessment is based on measurements of the difference in the pulse transit time and the distance travelled by the pulse between the two recording sites (right carotid and right femoral artery), with the overestimation of the true distance being corrected by multiplying the direct distance by 0.8. The aPWV is automatically calculated as the average of 8–10 transit times, detected with mechano-transducers placed at the carotid and femoral pulses.

Peripheral endothelial function was analyzed in the supine position via reactive hyperemia peripheral arterial tonometry (RH-PAT), using an EndoPAT2000 device (Itamar Medical Ltd., Caesarea, Israel), according to the manufacturer’s instruction, as described previously [36,37]. Briefly, a blood pressure cuff was placed on one upper arm (study arm), while the contralateral arm served as a control (control arm). PAT probes were placed on the second finger of each hand. After a 5 min equilibration period, the arm cuff was inflated to 200 mmHg for 5 min and then deflated to induce reactive hyperemia. RHI (reactive hyperemia index) reflecting the extent of post-ischemic reactive hyperemia is automatically calculated as the ratio of the average amplitude of the tonometry signal over 1 min, starting at 1.5 min after cuff deflation, divided by the average amplitude of the signal over a 2.5 min baseline period.

### 2.6. Biological Assays

Insulin and glucose measurements were, respectively, performed via an Elecsys assay on a COBAS 8000 e602 and a GLUC3 assay on a COBAS 8000 c701 (Roche Diagnostics, Meylan, France). Insulin resistance was assessed with the HOMA-IR, calculated as follows: [glucose (mmol/L) × insulin (UI/L)]/22.5.

Total cholesterol, HDL-cholesterol, and triglycerides levels were measured using an enzymatic method on a COBAS 8000 c502 (Roche Diagnostics, Meylan, France). LDL-cholesterol (LDL-C) was calculated using the Friedewald equation.

Erythrocyte vitamin E and total plasma vitamin C concentrations were measured by high-performance liquid chromatography coupled with UV detection (Acquity I-Class/FLR detector, Waters Corporation, Guynancourt, France).

### 2.7. Data Analysis

Data were expressed as the mean ± sem. Statistical analyses were conducted using R 4.3.3 (2024, The R Foundation for Statistical Computing, Vienna, Austria) and GraphPad Prism (V6.05, RRID: SCR_002798) and were performed blinded to the randomization group with either a *t*-test, repeated-measures two-way ANOVA, or mixed-effects model when missing values followed by Sidak’s comparison test, using the group, time, and subject as fixed factors and time × group as the interaction term. *p*-values < 0.05 were considered to be significant.

## 3. Results

### 3.1. Subject Characteristics

A total of 120 participants (mean age: 65.6 ± 04 years; 59 males and 61 females) completed the study. They were evenly distributed between the placebo and SpSi groups. At the end of the protocol, after 6 months of supplementation (D180), two participants from the placebo group and one from the SpSi group were removed from the study. As a result, at D180, 117 subjects completed the study: 58 in the placebo group and 59 in the SpSi group.

**Figure 1:**
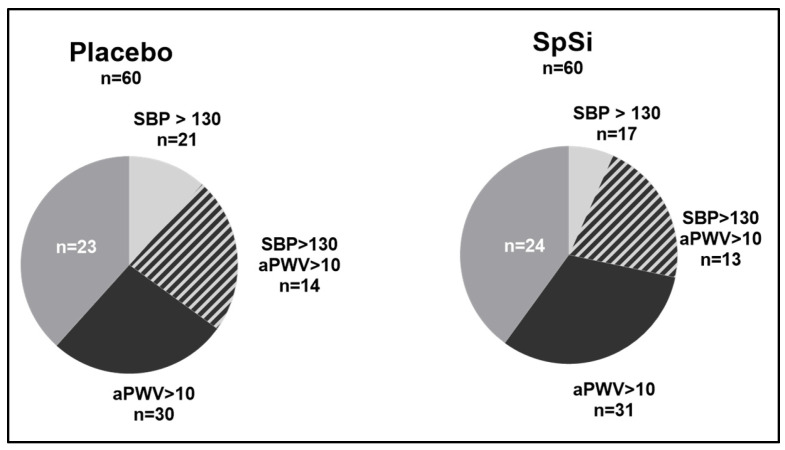
Distribution of subjects in the placebo and SpSi groups. This distribution is based on the following baseline criteria: systolic blood pressure (SBP) greater than or equal to 130 mmHg (SBP > 130; light grey area) and aortic pulse wave velocity higher than 10 m/s (aPWV > 10; black area). Some subjects meet both criteria (hatched area).

At baseline, the two groups were similar (Table 1) and, overall, consisted of healthy subjects with normal BMI levels, blood pressure, carbohydrate and lipid metabolisms, and antioxidant status (vitamin C and vitamin E). Additionally, we noted that some subjects in both groups had high–normal systolic blood pressure (SBP) (SBP between 130 and 150 mmHg) [38] and/or an aortic pulse wave velocity higher than 10 m/s [39]. Considering these parameters, the distribution of subjects was equivalent in the two groups, supplemented (SpSi) and not supplemented (placebo) (Figure 1), which allowed for subsequent subgroup analysis.

### 3.2. Effect of SpSi Supplementation in All Subjects

**Figure 2:**
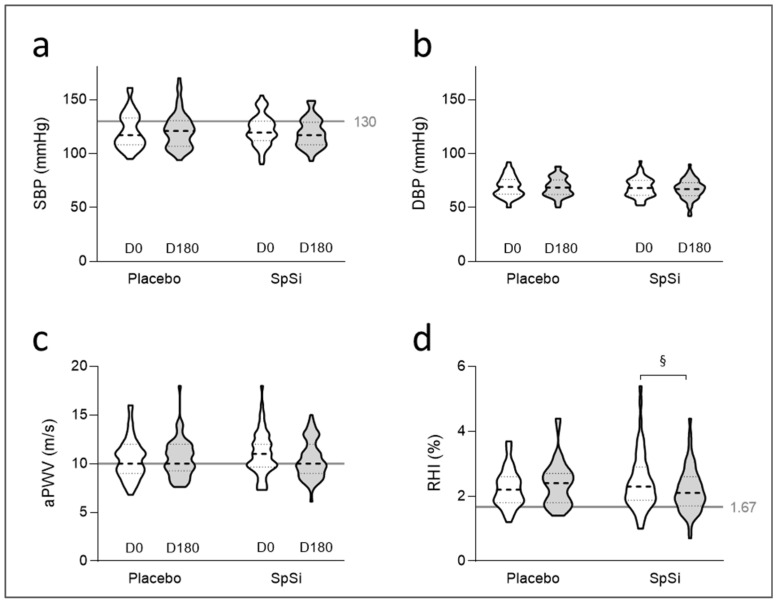
Effects of SpSi supplementation on vascular parameters for all subjects who completed the study. Violin plots show distributions using density curves of systolic (SBP) (**a**) and diastolic (DBP) blood pressures (**b**), aortic pulse wave velocity (aPWV) (**c**), and reactive hyperemia index (RHI) (**d**) in the placebo and SpSi groups before (D0) and after 6 months of SpSi supplementation (D180). The grey lines highlight the normal limit values: 130 mmHg for SBP, 10 m/s for aPWV and 1.67 for RHI. Data were analyzed using a mixed-effects model followed by Sidak’s multiple-comparisons test, with *n* = 60 for the two groups. ^§^ *p* < 0.05 for comparison between D0 and D180.

After 6 months of supplementation, we found no change in the BP and aPWV in both groups (Table 2, Figure 2a,c). However, we observed a slight decrease in the RHI in the SpSi group. Although statistically significant for SpSi, this variation is modest and not clinically relevant (8.9 ± 3.6% decrease in the RHI value at D0). No differences were observed in carotid intima–media thicknesses (Table 2), and the biological parameters remained within normal limits.

### 3.3. Effects of SpSi Supplementation in Subjects with Elevated Aortic Pulse Wave Velocity

**Figure 3:**
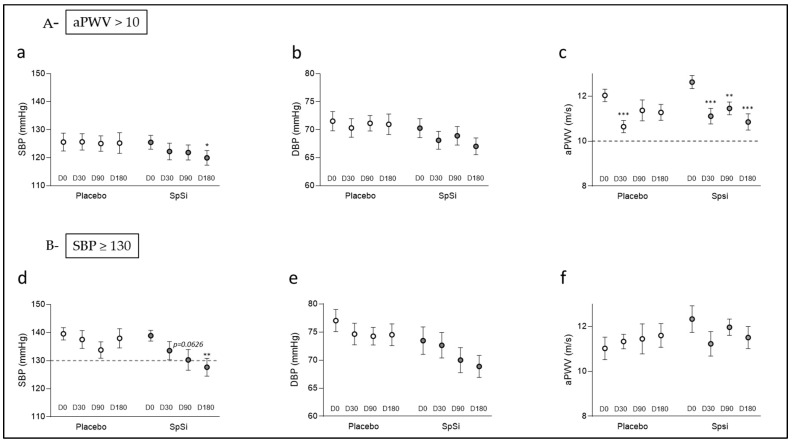
Evolution of vascular parameters in subgroup populations during SpSi supplementation. The graphs represent the evolution of the SBP (**a**,**d**), DBP (**b**,**e**), and aPWV (**c**,**f**) in the subgroup SBP ≥ 130 mmHg (**A**) and the subgroup with aPWV > 10 m/s (**B**). Data represent the mean ± sem at D0, D30, D90, and D180 and were analyzed using a mixed-effects model followed by Sidak’s multiple-comparisons test. * *p* < 0.05; ** *p* < 0.01; and *** *p* < 0.001 for comparison with D0 values.

We identified subjects with a baseline aPWV above 10 m/s: 30 subjects in the placebo group and 31 subjects in the SpSi group (51.7% and 50%, respectively) (Figure 1). The men/women ratio differed between the placebo and SpSi, being 56.6% and 43.3%, respectively (Table 3). At D0, all parameters were similar in the two subgroups. After 6 months, the aPWV and SBP were significantly reduced in the SpSi group. In this group, both the aPWV and SBP showed a progressive decrease from D0 to D180, as illustrated in Figure 3. At D30, both groups exhibited a drop in the aPWV. Then, the aPWV remained low in SpSi at D90 and D180, while it returned to baseline values for the placebo group (Figure 3c). The reduction in the aPWV occurred in 22 SpSi subjects (with an average of 13.3 ± 2.9%; Figure 3c *p* < 0.0001) and was normalized (i.e., <10 m/s) in 16 subjects. The DBP slightly tended to decrease (*p* = 0.0538) (Figure 3e). The fall in the SBP averaged 5.5 ± 2.1 mmHg or 4.1 ± 1.8% of the baseline value. A reduction in the SBP was noted in 22 out of 32 subjects, even when the baseline value did not exceed the cutoff value of 130 mmHg. In subjects with baseline high–normal SBP (*n* = 13), we noted a significant decrease (−7.8 ± 2.1% *p* = 0.0037) after 6 months of supplementation. In the subgroup with both a high–normal BP and high aPWV (aPWV + SBP), the aPWV did not significantly differ after SpSi supplementation. We also evidenced a slight reduction in the RHI for the SpSi group at D180, corresponding to an average 9.2 ± 4.6% of the baseline value (*p* = 0.0192, Table 3), which appeared not clinically relevant. No differences were observed for biological parameters.

### 3.4. Effects of SpSi Supplementation in Subjects with Elevated Blood Pressure

Interestingly, 21 subjects in the placebo group and 17 in the SpSi group had an elevated SBP (>130 mmHg) (representing, respectively, 35% and 28% of the subjects) (Figure 1). At D0, the two subgroups were similar, except for the men/women ratio (Table 4), with a higher rate of men with a high–normal BP in the placebo group (66.7% vs. 35.3%). In the SpSi group, the SBP decreased over time (Figure 3d), with values differing from the baseline at 90 and 180 days of supplementation. After 6 months (D180), the SBP in the SpSi group showed a significant decrease over time (T, *p* = 0.007) along with notable interaction effects (G × T, *p* = 0.0202). Additionally, there was a slight trend observed toward a decrease in the pulse pressure (PP) and diastolic pressure (DBP), with *p*-values of 0.0530 and 0.0722, respectively (Table 4). SBP reduction was observed in 13 subjects out of 17 (76.4%), including 9 (53%) for whom the SBP normalized (below the cutoff value of 130 mmHg). The average decrease is 11.2 ± 2.2 mmHg and represents 8.2 ± 1.6% of the initial SBP. This rate is similar to that observed for the subgroup with aPWV > 10 m/s. DBP variation over time tended to show a decrease, which was not observed in the placebo group (Figure 3e). We observed a DBP reduction in 11 out of 17 subjects at D180, which was, on average, 6.6 ± 2.9 mmHg (9.2 ± 4.7%) (Table 4). In the placebo group, 15 out of 21 subjects (71%) still had an SBP above 130 mmHg at D180. In this group, the non-significant fluctuations in the SBP and DBP between D0 and D180 (Figure 3d,e) were 1.6 ± 2.8 mmHg and 3.2 ± 1.9 mmHg, respectively, representing 2.9 ± 5.1% and 1.8 ± 2.8% of the initial values. The aPWV, otherwise within the normal range, was unchanged (Table 4, Figure 3f). No change in the biological parameters was observed in this subgroup either.

## 4. Discussion

The present study aimed to evaluate the effect of 6 months of dietary supplementation with silicon-enriched spirulina (SpSi) on arterial wall properties, specifically focusing on arterial stiffness and blood pressure in an elderly population. Our research indicates that while SpSi supplementation did not significantly affect the general population, it resulted in a notable decrease in the arterial pulse wave velocity (aPWV) and systolic blood pressure (SBP) among specific subgroups of elderly individuals with elevated baseline values.

Participants with an elevated aortic pulse wave velocity (aPWV greater than 10 m/s) experienced an average reduction of 13.3% with SpSi supplementation. This reduction was also associated with a significant decrease in the SBP among individuals who had high–normal blood pressure. The observed decrease in aPWV is an important finding, as an elevated aPWV is a recognized indicator of arterial stiffness and an independent predictor of cardiovascular events in older adults [11,40].

In individuals with high systolic blood pressure (SBP > 130 mmHg), supplementation with SpSi led to a significant reduction in their SBP over a period of 6 months, with an average decrease of 11.2 mmHg (8.2%). Notably, 53% of these individuals experienced a substantial reduction in their SBP to a healthy level. This finding is particularly important, as there is a strong link between high SBP and cardiovascular events in older adults [41,42]. Although the trends observed were not statistically significant, there was a noticeable decrease in the diastolic blood pressure (DBP) and pulse pressure (PP). This finding aligns with the positive effects of SpSi on overall blood pressure regulation. An increase in pulse pressure is associated with age-related arterial stiffness and indicates a decline in arterial compliance [2]. Therefore, a reduction in pulse pressure may suggest improved arterial function and more effective management of blood pressure.

These findings suggest that SpSi supplementation may help alleviate arterial stiffness, thereby potentially reducing cardiovascular risk in elderly populations.

Our study’s results align with previous research indicating that silicon supplementation has a positive impact on vascular health. For instance, several studies have shown that dietary silicon can improve the structural integrity of connective tissues and reduce the risk of atherosclerosis in both human and animal models [21,22,23,43]. Additionally, it has been shown that silicon-enriched spirulina can improve early markers of atherosclerosis in hamsters [26] and lower blood pressure in hypertensive rats [27]. Our study builds on these findings and goes further by examining a healthy, elderly human population, emphasizing the potential role of silicon supplementation in preventing the cardiovascular decline associated with aging.

The positive effects of SpSi supplementation can be partly attributed to the biological role of silicon in preserving the structural integrity and elasticity of connective tissues [44,45]. Silicon is essential for the synthesis and stabilization of collagen and elastin fibers, which are vital for maintaining the elasticity of arteries [44,46]. Arterial stiffness is a significant indicator of cardiovascular aging. Decreased silicon levels, which paralleled changes in elastic fibers, have been observed with the development of atherosclerosis [25,47]. The presence of silicon in SpSi may help maintain arterial flexibility by supporting the organization of collagen and elastin. Furthermore, spirulina, known for its antioxidant and anti-inflammatory properties, may also contribute to these benefits. Research has shown that spirulina positively affects vascular function and reduces oxidative stress and inflammation, with both of them playing a role in arterial stiffness and hypertension [30,48]. Therefore, the combined effects of metabolized silicon and spirulina could provide a synergistic approach to alleviating age-related cardiovascular health. Mineral-enriched spirulina is a versatile and sustainable tool for improving nutritional status and addressing mineral deficiencies. Spirulina is capable of bioaccumulating and metabolizing elements and nutrients. As a consequence, spirulina biomass becomes enriched with organic forms of elements and nutrients. This ability is considered in the cultivation of spirulina in order to enrich their primary biological value and their efficiency as diet supplements [49,50] and has proven its efficiency for zinc, selenium, or iron supplementation [51]. Spirulina supplements are widely used due to their GRAS status (Generally Recognized As Safe) and the clinically proven absence of health risks [52]. No toxic effects have been reported with silicon-enriched spirulina supplementation in rats [32], even at high doses exceeding the recommended daily intake of 6 g/day [53].

The results suggest that supplementing with the specific SpSi we used may effectively support healthy aging by preserving arterial elasticity and reducing blood pressure, particularly in older adults with elevated SBP or aPWV. As we age, blood pressure tends to increase [54,55], and aging blood vessels can make individuals more susceptible to conditions such as high blood pressure, atherosclerosis, and other cardiovascular diseases. Given the rising prevalence of high blood pressure and stiffening arteries among older populations, it is crucial to focus on preventing the progression of these conditions through healthy lifestyle choices [39,56,57]. Therefore, interventions aimed at addressing these modifiable risk factors could offer significant public health benefits.

The study has shown promising results, but it also has several limitations that need to be addressed. Firstly, the sample size, especially within the subgroups, was relatively small, which may restrict the generalizability of the findings. Secondly, the study did not take into account all potential confounding factors, such as diet, physical activity, and other lifestyle variables, that could influence BP and arterial stiffness. Both BP and aPWV are known to be sensitive to lifestyle changes, including dietary modifications and physical activity [58]. Short-term dietary changes—like reduced sodium or alcohol intake—can have immediate effects on BP, while the aPWV primarily reflects more chronic changes in arterial stiffness [59]. In our study, both the BP and aPWV decreased following supplementation, indicating that if any lifestyle changes did occur, they were not the main factors driving these effects. Additionally, biological parameters in our population, including lipid, glucose, and insulin blood levels and antioxidant enzymes, were within normal ranges and showed no significant variations. This suggests that the observed benefits of SpSi supplementation on arterial function are unlikely to be attributed to metabolic changes. Nonetheless, lifestyle factors should still be considered as potential contributors.

Lastly, the study duration of 6 months, while adequate to observe some effects, may not be long enough to capture the long-term benefits or risks associated with ongoing SpSi supplementation.

Furthermore, the study did not distinguish between the effects of silicon and those of spirulina, making it challenging to identify the individual contributions of each component. Previous research, however, demonstrated that the benefits of SpSi supplementation on blood pressure in spontaneously hypertensive rats could be attributed to silicon enrichment, since natural spirulina supplementation alone showed no significant effects [25]. To better understand the distinct roles of these components, future studies should investigate them separately.

Further research involving larger populations, extended follow-up periods, and controlled conditions is essential to confirm these results and explore the potential of SpSi as a preventive intervention for age-related cardiovascular diseases. Further studies should also focus on determining the optimal dosing, duration, and form of silicon to maximize its bioavailability and effectiveness across different populations.

## 5. Conclusions

This study indicates that consuming this specific silicon-enriched spirulina may help lower the systolic blood pressure and reduce arterial stiffness in healthy elderly individuals with high initial values. This could represent a new approach to decreasing the cardiovascular risks associated with aging. While these findings are promising, additional research is necessary to fully understand the underlying mechanisms, long-term effects, and practical applications of silicon-enriched spirulina supplementation for promoting healthy aging.

## Figures and Tables

**Table 1:** Baseline physiological parameters for all subjects in each group (placebo and SpSi).

Variable	Placebo (*n* = 60)	SpSi (*n* = 60)	*p*-Value
Age (year)	65.9 ± 0.6	65.3 ± 0.5	0.6169
BMI (kg/m^2^)	24.2 ± 0.5	24.5 ± 0.4	0.3728
Systolic blood pressure (mmHg)	121.8 ± 1.8	120.8 ± 2.1	0.4455
Diastolic blood pressure (mmHg)	68.3 ± 1.1	69.3 ± 1.2	0.5537
Heart rate (bpm)	61.3 ± 1.5	62.1 ± 1.2	0.3721
Aortic pulse wave velocity (m/s)	10.48 ± 0.27	11.01 ± 0.29	0.1565
Reactive hyperemia index	2.27 ± 0.08	2.46 ± 0.11	0.4055
Hemoglobin	14.24 ± 0.14	14.23 ± 0.14	0.9646
Glucose (mmol/L)	5.57 ± 0.07	5.43 ± 0.07	0.1606
Insulin (pmol/L)	72.2 ± 6.1	64.5 ± 4.2	0.4441
HOMA-IR	3.34 ± 0.69	2.27 ± 0.16	0.2886
Total cholesterol (mmol/L)	5.70 ± 0.11	5.55 ± 0.11	0.3141
HDL-cholesterol (mmol/L)	1.80 ± 0.07	1.81 ± 0.06	0.6939
LDL-cholesterol (mmol/L)	3.41 ± 0.10	3.22 ± 0.09	0.1117
Triglycerides (mmol/L)	1.09 ± 0.05	1.19 ± 0.07	0.5224
Vitamin C (µmol/L)	67.5 ± 3.2	59.9 ± 3.2	0.2143
Vitamin E (µg/L)	0.52 ± 0.05	0.66 ± 0.08	0.4087

BMI: body mass index; HOMA-IR: homeostasis model assessment of insulin resistance.

**Table 2:** Physiological and vascular parameters measured at baseline (D0) and after 6 months of supplementation (D180) for all subjects at 6-month follow-up and for both placebo and SpSi.

Variable	Placebo			SpSi		
	D0	D180	*p*-Value	D0	D180	*p*-Value
Subjects (*n*)	58			59		
Men (*n*)	28			28		
Women (*n*)	30			31		
Age (y)	65.2 ± 0.9			66 ± 0.6		0.6169 #
BMI (kg/m^2^)	24.6 ± 0.5			24.1 ± 0.5		0.6241 #
Systolic blood pressure (mmHg)	120.8 ± 2.1	121.5 ± 2.1	0.8995	121.7 ± 1.8	118.9 ± 1.7	0.1165
Diastolic blood pressure (mmHg)	69.6 ± 1.2	68.8 ± 1.1	0.8302	68.2 ± 1.1	67.0 ± 1.2	0.4589
Mean blood pressure (mmHg)	95.2 ± 1.5	95.2 ± 1.5	>0.9999	95 ± 1.3	92.9 ± 1.2	0.1423
Pulse pressure (mmHg)	51.2 ±1.6	52.6 ±1.6	0.5892	53.5 ±1.6	51.9 ±1.7	0.4269
aPWV (m/s)	10.5 ± 0.3	10.5 ± 0.2	0.9987	11.0 ± 0.3	10.4 ± 0.2	0.0864
RHI (%)	2.25 ± 0.08	2.39 ± 0.09	0.2989	2.43 ± 0.11	2.19 ± 0.09	0.0437
Right carotid IMT (mm)	0.72 ± 0.01	0.70 ± 0.01	0.3514	0.73 ± 0.01	0.71 ± 0.02	0.2208
Left carotid IMT (mm)	0.76 ± 0.02	0.74 ± 0.01	0.2228	0.75 ± 0.02	0.74 ± 0.01	0.9398

Values are presented as the mean ± sem, and the *p*-value was determined using either the Mann–Whitney test between the placebo and SpSi at D0 (#) or using a mixed-effects model followed by Sidak’s multiple-comparisons test between D0 and D180. BMI: body mass index; aPWV: aortic pulse wave velocity; RHI: reactive hyperemia index; IMT: intima–media thickness.

**Table 3:** Physiological and vascular parameters at D0 and after 6 months of supplementation (D180) in subpopulation with aPWV > 10 m/s at baseline.

Variable	Placebo (aPWV > 10)			SpSi (aPWV > 10)		
	D0	D180	*p*-Value	D0	D180	*p*-Value
Subjects (*n*)	28			28		
Men (*n*)	17			12		
Women (*n*)	12			16		
Age (y)	65.7 ± 0.7			66.5 ± 0.9		0.6710 #
BMI (kg/m^2^)	24.7 ± 0.6			24.3 ± 0.7		0.3111 #
Systolic blood pressure (mmHg)	126.8 ± 3.1	125.1 ± 3.5	0.7482	125.5 ± 2.5	119.9 ± 2.6	0.0233
Diastolic blood pressure (mmHg)	72.1 ± 1.6	70.7 ± 1.8	0.6491	70.2 ± 1.7	67.9 ± 1.5	0.0538
Mean blood pressure (mmHg)	99.4 ± 2.1	97.9 ± 2.5	0.6211	97.8 ± 1.8	94.3 ± 1.6	0.0318
Pulse pressure (mmHg)	54.8 ±2.3	54.4 ±2.5	0.9908	55.3 ±2.3	52.9 ±2.4	0.3677
aPWV (m/s)	12.1 ± 0.3	11.4 ± 0.3	0.0920	12.6 ± 0.3	10.9 ± 0.4	<0.0001
RHI (%)	2.36 ± 0.11	2.31 ± 0.10	0.8642	2.54 ± 0.10	2.20 ± 0.13	0.0192
Right carotid IMT (mm)	0.72 ± 0.02	0.71 ± 0.02	0.8156	0.74 ± 0.02	0.73 ± 0.03	0.6998
Left carotid IMT (mm)	0.76 ± 0.02	0.76 ± 0.02	0.9023	0.75 ±0.02	0.75 ± 0.02	0.9796

Values are presented as the mean ± sem, and the *p*-value was determined using either the Mann–Whitney test between the placebo and SpSi at D0 (#) or using a mixed-effects model followed by Sidak’s multiple-comparisons test between D0 and D180. BMI: body mass index; aPWV: aortic pulse wave velocity; RHI: reactive hyperemia index; IMT: intima–media thickness.

**Table 4:** Physiological and vascular parameters at D0 and after 6 months of supplementation (D180) in subpopulation with SBP ≥ 130 mmHg at baseline (D0) for both placebo and SpSi.

Variable	Placebo (SBP ≥ 130)			SpSi (SBP ≥ 130)		
	D0	D180	*p*-Value	D0	D180	*p*-Value
Subjects (*n*)	20			17		
Men (*n*)	14			6		
Women (*n*)	6			11		
Age (y)	66.1 ± 1.0			67.9 ± 1.1		0.1859 *
BMI (kg/m^2^)	24.8 ± 0.7			23.8 ± 0.9		0.2344 *
Systolic blood pressure (mmHg)	139.5 ± 2.1	137.1 ± 3.3	0.7482	138.9 ± 1.9	127.6 ± 3.2	0.0004
Diastolic blood pressure (mmHg)	76.9 ± 1.9	73.9 ± 1.9	0.6491	73.5 ± 2.4	68.9 ± 2.0	0.0721
Mean blood pressure (mmHg)	108.2 ± 1.9	105.5 ± 2.6	0.6211	106.2 ± 1.7	98.3 ± 2.1	0.0005
Pulse pressure (mmHg)	62.6 ±2.4	63.2 ±2.8	0.9908	65.4 ± 2.7	58.7 ± 3.3	0.0530
aPWV (m/s)	11.2 ± 0.5	11.7 ± 0.5	0.0920	12.3 ± 0.6	11.5 ± 0.5	0.3726
RHI (%)	2.5 ± 0.2	2.3 ± 0.1	0.8642	2.7 ± 0.3	2.5 ± 0.2	0.7084
Right carotid IMT (mm)	0.75 ± 0.03	0.75 ± 0.02	0.8156	0.74 ± 0.02	0.75 ± 0.03	0.3868
Left carotid IMT (mm)	0.82 ± 0.03	0.79 ± 0.03	0.9023	0.74 ±0.03	0.74 ± 0.03	0.9980

Values are presented as the mean ± sem, and the *p*-value was determined using either the Mann–Whitney test between the placebo and SpSi at D0 (*) or using a mixed-effects model followed by Sidak’s multiple-comparisons test between D0 and D180. BMI: body mass index; aPWV: aortic pulse wave velocity; RHI: reactive hyperemia index; IMT: intima–media thickness.

## Data Availability

The data presented in this manuscript may become available upon request to the corresponding author.
